# The effect of cattle-administered ivermectin and fipronil on the mortality and fecundity of *Anopheles arabiensis* Patton

**DOI:** 10.1186/s13071-021-04846-8

**Published:** 2021-07-02

**Authors:** Takalani I. Makhanthisa, Leo Braack, Heike Lutermann

**Affiliations:** 1 Mammal Research Institute, Department of Zoology & Entomology, University of Pretoria, Pretoria, South Africa; 2 UP Institute for Sustainable Malaria Control, Faculty of Health Sciences, University of Pretoria, Pretoria, South Africa; 3 Faculty of Tropical Medicine, Malaria Consortium, Mahidol University, Bangkok, Thailand

**Keywords:** Malaria, Vector control, Endectocides, Ivermectin, Fipronil, *Anopheles arabiensis*, Cattle, Livestock

## Abstract

**Background:**

Malaria control primarily depends on two vector control strategies: indoor residual spraying (IRS) and long-lasting insecticide-treated nets (LLINs). Both IRS and LLIN target indoor-biting mosquitoes. However, some of the most important malaria vectors have developed resistance against the chemical compounds used in IRS and LLINs. Insecticide-induced behavioural changes in vectors, such as increased outdoor feeding on cattle and other animals, also limit the effectiveness of these strategies. Novel vector control strategies must therefore be found to complement IRS and LLINs. A promising tool is the use of cattle-applied endectocides. Endectocides are broad-spectrum systemic drugs that are effective against a range of internal nematodes parasites and blood-feeding arthropods. The aim of this study was to investigate the effect of two endectocide drugs, injectable ivermectin and topical fipronil, on the survival and fecundity of zoophilic *Anopheles arabiensis.*

**Methods:**

Laboratory-reared mosquitoes were allowed to feed on cattle treated with either injectable ivermectin (0.2 mg/kg), topical fipronil (1.0 mg/kg) or saline (control) on days 0, 1, 4, 7, 13, 21 and 25 post-treatment, and mortality and egg production were recorded daily.

**Results:**

Compared to controls, the mortality of *An. arabiensis* increased by 3.52- and 2.43-fold with injectable ivermectin and topical fipronil, respectively. The overall fecundity of mosquitoes that fed on both ivermectin- and fipronil-treated cattle was significantly reduced by up to 90 and 60%, respectively, compared to the control group. The effects of both drugs attenuated over a period of 3 weeks. Injectable ivermectin was more effective than topical fipronil and increased mosquito mortality by a risk factor of 1.51 higher than fipronil. Similarly, both drugs significantly reduced the fecundity of *An. arabiensis*.

**Conclusions:**

This study demonstrates that injectable ivermectin and topical fipronil are able to suppress *An. arabiensis* density and could help to reduce outdoor malaria transmission. Data from the present study as well as from other similar studies suggest that current-generation endectocides have a limited duration of action and are expensive. However, new-generation, sustained-release formulations of ivermectin have a multi-week, high mortality impact on vector populations, thus holding promise of an effective reduction of outdoor malaria transmission.

**Graphical abstract:**

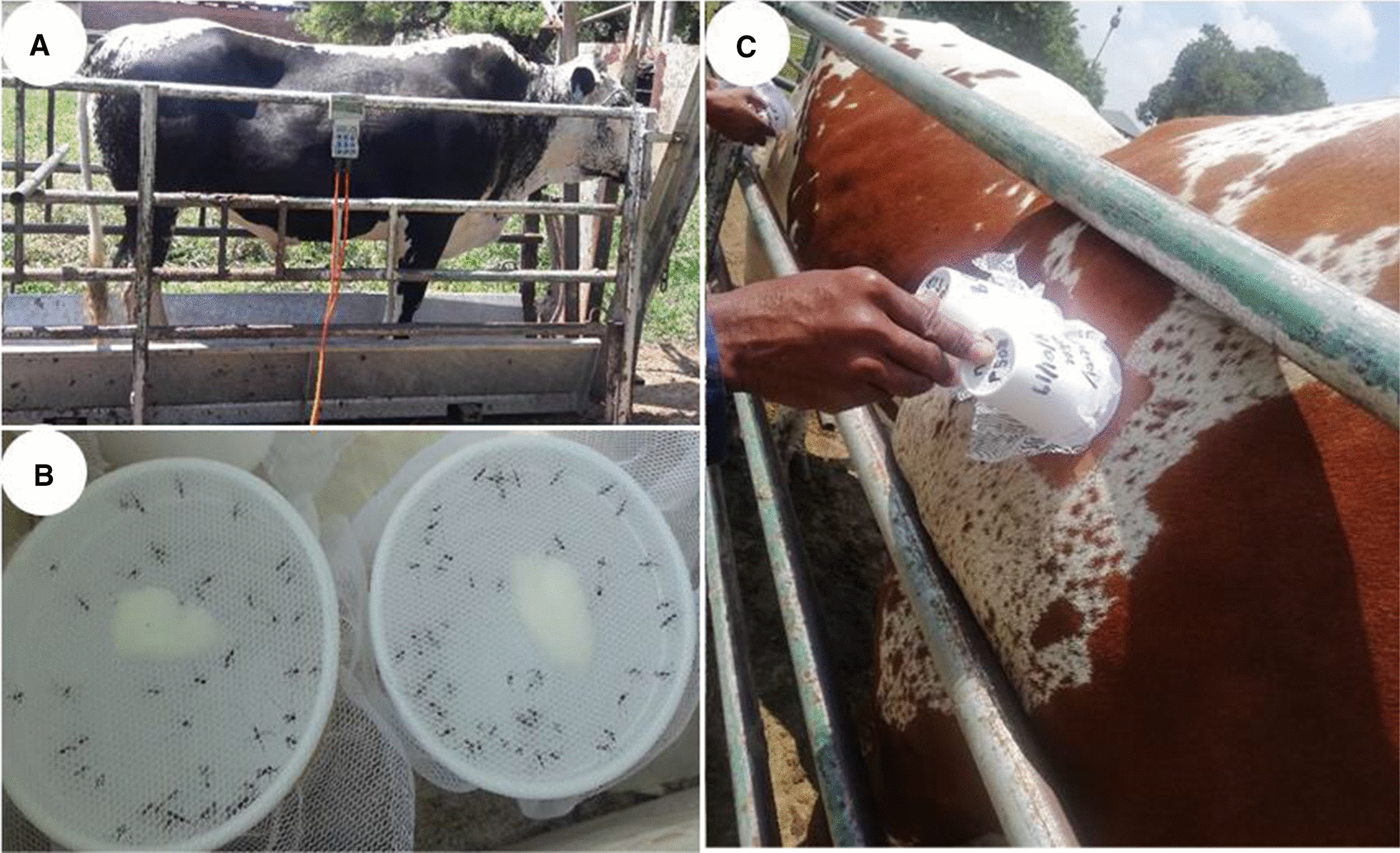

## Background

Malaria is a preventable and treatable disease that infects millions of people globally every year. The World Health Organisation reported the global malaria burden for 2018 as an estimated 228 million cases associated with approximately 405,000 deaths [1]. This constitutes a significant increase from the 219 million cases reported for 2017 [2]. Africa is the most malaria-burdened continent, accounting for 93% of reported cases in 2018 [1]. Following notable declines in global malaria indices between 2000 and 2015, these gains have reached a plateau, and malaria cases have once again increased although mortality has declined [1–3]. South Africa has shown similar trends, with significant decreases over the past two decades [4]. In the 1999/2000 malaria season, there were over 60,000 malaria cases, decreasing to less than 13,000 in the 2013/2014 malaria season [5]. However, a steep increase in malaria cases and associated mortality in recent years (2017/2018) has revealed the fragile nature of control efforts and the ease with which malaria can resurge [2, 3]. This multi-year trend of stagnating malaria control is an indication that the current malaria control strategies are no longer adequate and that new and/or supplementary measures must be developed.

Human malaria is caused by five *Plasmodium* parasites that are transmitted by females of certain *Anopheles* mosquitoes [6]. In Africa, malaria vector species are mainly from two taxonomic clusters: the *Anopheles gambiae* complex and the *Anopheles funestus* group [7]. The most significant species from the *An. gambiae* complex are *An. arabiensis* and *An. gambiae*, and *An. funestus* is the most significant species from the *An. funestus* group [8, 9]. However, a number of other, less efficient secondary vectors are also capable of transmitting malaria [10]. The *An. funestus* group is broadly distributed across Africa, with its major species, *An. funestus*, widely distributed over subtropical and tropical Africa where it breeds in permanent large water bodies with emergent vegetation [7, 11]. *Anopheles funestus* is highly anthropophilic (human biting) and exhibits endophilic (indoors) feeding and resting behaviours [7, 12]. The major malaria vector species from the *An. gambiae* complex, *An. arabiensis* and *An. gambiae*, are also widely distributed across Africa [13]. Members of the *An. gambiae* complex prefer to breed in temporary bodies of water that are clean and shallow [14, 15]. While *An. gambiae* is highly anthropophilic [12], *An. arabiensis* is zoophagic, feeding readily on animals in most areas, particularly cattle [15–17]. Studies have shown that *An. arabiensis* prefers to feed outdoors even in areas where it mostly feeds on humans [18, 19]. *Anopheles arabiensis* is therefore less impacted by indoor vector control strategies [16]. The different behaviours of the major malaria vectors are a challenge in terms of malaria transmission control [20]. Recently, there has been increasing evidence of plasticity in the feeding behaviours of malaria vectors which is also affected by the availability of different host types [21]. The plasticity would serve as an advantage for malaria control strategies as alternative hosts that are abundant could be used for targeted vector control. In South Africa, *An. arabiensis* is widely acknowledged as the main vector although other species, such as *An. merus*, *An. rivulorum* and *An. funestus*, may be important at the local level [22].

The primary control of malaria for decades in most malaria-endemic regions of the world has been the implementation of indoor residual spraying (IRS) and long-lasting insecticide-treated bednets (LLINs) [23, 24]. LLINs are primarily treated with pyrethroid insecticides, IRS programmes involve the application of a range of pyrethroids, organophosphates, carbamates as well as the organochloride dichlorodiphenyltrichloroethane (DDT) to internal walls and ceilings of housing structures [25]. Both pyrethroid insecticides and DDT have the same target on the voltage-gated sodium channel found on mosquitoes’ neurons; therefore, vectors can become resistant to both strategies [26]. Furthermore, these resistance alleles have spread at a rapid rate throughout Africa, requiring urgent action to prevent an increase in malaria [25, 27]. The increasing resistance that has developed within various *Anopheles* species to these insecticides poses a major challenge to the effectiveness of these key vector control methods [20, 25, 28].

Aside from escalating insecticide resistance affecting the value of IRS and LLINs as indoor interventions, additional challenges are emerging [29]. The major malaria vector species historically preferred feeding indoors [30, 31]. However, some recent studies have shown a shift in the behaviours of *An. gambiae* and *An. funestus* to feeding outdoors instead of indoors in some areas where IRS and LLIN programmes have been implemented [30, 32]. Zoophilic characteristics in some of the major vectors are also a challenge [15]. Yet another challenge is a temporal shift in feeding behaviour, with the malaria vector species biting in the early evenings and mornings when people are not under their protective nets [33, 34]. Given these limitations of IRS and LLINs to curb transmission, the challenge of residual malaria poses a serious hurdle in reaching malaria elimination objectives, and the number of malaria cases and malaria-related deaths remain unacceptably high [35].

The use of cattle-administered endectocides is a promising strategy for outdoor vector control that could complement IRS and LLINs [36, 37]. Several endectocide drugs are effective against a wide range of both endo- and ectoparasitic nematodes and arthropods in humans and cattle [20, 36], including ivermectin, eprinomectin, fipronil and diflubenzuron [38]. Ivermectin was the first endectocide to be used in humans and continues to be used to treat river blindness through mass drug administration (MDA) [37, 39]. Ivermectin is a lipophilic drug belonging to the avermectin class of macrocyclic compounds [40] and is also used to treat onchocerciasis, strongyloidiasis, lymphatic filariasis, scabies and head lice [20, 39]. Endectocides are also of veterinary importance as they are used to control parasites in animals such as cattle and goats [41].

Endectocides utilise a different mode of action against insects to that of IRS and LLIN [36] and can thus complement traditional control measures. Yakob et al. [42] conducted the first study that examined the combined use of endectocide-treated livestock with LLINs to enhance malaria control. Through simulation, the study showed that targeting livestock-biting behaviour for controlling malaria mosquitoes has potentially excellent synergy with LLINs to decrease malaria prevalence [42]. In the vector, ivermectin primarily targets the glutamate gated chloride channels, which are neurotransmission inhibitors through their 16-membered macrocyclic lactone [20, 43–45]. Binding to the channels leads to an influx in chloride ions that in turn leads to neuromuscular junction dysfunction and hyperpolarisation [46]. In contrast, fipronil is a phenylpyrazole compound that works by blocking the GABA-gated ion channels, which are also in the central nervous system of arthropods [46–48]. Exposure to both ivermectin and fipronil results in flaccid paralysis and eventually death in the target parasites [20, 47]. These chloride gated iron channels are not present in vertebrates; therefore, ivermectin and fipronil are non-toxic to humans and livestock [49]. Ivermectin MDA in humans is a promising malaria control tool that targets mosquitoes with control-avoidance biting behaviours and those that have developed physiological insecticide resistance [36, 45]. Several studies have shown that ivermectin-treated human blood decreases the survival, feeding frequency, blood-meal digestion and fecundity of mosquitoes [50–52]. Ivermectin (brand name Mectizan®) has been used successfully in humans for river blindness since 1987 and does not have toxic side effects at recommended doses [53, 54]. Several studies have shown that ivermectin and several other endectocides applied to cattle or other livestock also decrease the survival and fecundity of malaria vector mosquitoes [16, 36, 49, 55]. Fipronil has been approved for use on domestic animals in many countries and is used to control arthropods such as ticks, cockroaches and fleas [56]. In addition, fipronil has been used in cattle to control leishmaniasis vectors [57, 58]. Similarly, fipronil is effective against all life stages of *Anopheles* mosquitoes [16]. However, field studies on its use against mosquitoes are limited.

The effectiveness of endectocides is linked to their pharmacokinetics, which vary across different species [59]. The route of administration also has a significant effect on the pharmacokinetics of endectocides [60]. Ivermectin pharmacokinetics studies have been conducted in cattle to compare subcutaneous and oral routes of administration [16, 61]. One study showed that ivermectin injected subcutaneously in cattle was effective against *An. arabiensis* mosquitoes for a longer period of time than was oral or topical treatment [16]. In another study higher ivermectin plasma concentrations were produced with subcutaneous treatment than with oral administration [61]; high concentrations produce an enhanced systemic availability which results in higher efficacy against the targeted parasites. For formulations typically used in recent years, the maximum concentration of subcutaneously injected ivermectin was reached at day 1 while the minimum was reached after 25 days [61]. Similarly, fipronil injected in cattle reached its maximum and minimum concentrations rapidly within 24 h [62]. In a study where the pour-on fipronil formulation was administered in cattle to investigate its effect against ticks, the mean plasma concentration values over time varied, with the maximum concentration of 73.7 g/l reached after 2.5 days [63]. The pour-on fipronil concentration reached its half-life at day 19 and decreased slowly until its minimal level at day 40 [63]. Topical treatment results in exposure to environmental degradation, such as mechanical removal by rain [62]. Factors such as body weight, nutrition type and physiological status also lead to variation in drug concentrations within individuals of the same species [59].

Currently there are no studies investigating the impact of cattle-administered endectocides on mosquitoes in South Africa. Studies that have conducted this type of research in other countries have mostly focussed on ivermectin only. The present study included an additional potential endectocide, fipronil. This study also considered the pharmacokinetics profiles of the two drugs and conducted feeding trials at various points, including at the time of minimum and maximum concentrations. The aim of this study was to investigate the effectiveness of two endectocides, namely ivermectin and fipronil, for control of *An. arabiensis* in South Africa. The specific objectives were: (i) to demonstrate that ivermectin and fipronil reduce adult survival and fecundity of *An. arabiensis*; (ii) to compare the efficacy of injectable ivermectin against that of topical fipronil; and (iii) to assess the duration efficacy of each endectocide. We predicted that endectocide treatment would result in a significant increased mortality of *An. arabiensis* and a reduction in the egg production of this mosquito species. We also predicted that injected ivermectin would be more effective than pour-on fipronil. We further predicted that the efficacy of both endectocides would last for 1 month, a prediction based on the manufacturer’s instructions regarding the duration of their effect against other parasites and data from previous related studies.

## Methods

### Insectary-rearing *An. arabiensis* mosquitoes

*Anopheles arabiensis* eggs were obtained from the Vector Control Laboratory of the South African National Institute for Communicable Diseases in Johannesburg, South Africa. Colonies of *An. arabiensis* were established and maintained in an insectary at the University of Pretoria, Faculty of Health Sciences. The insectary is kept at a constant temperature of 25 ± 2 °C, 75 ± 5% humidity and has a 12 h light:11 h darkness photoperiod. Eggs were placed in containers (2–5 l) filled with water and the larvae fed a mixture of powdered dog biscuits and yeast mixture at a ratio of 75:25. Growing larvae were subdivided into separate containers with water and allowed to mature. Mesh-netting covers over the larval basins prevented emerging adults from escaping. A small slit was cut into each cover to allow access for suction capture of adults daily. A mouth aspirator was used to transfer emerged adults into bucket-cages (5–20 l). A circular hole was made on the side of each bucket and fitted with a netting sleeve to allow the transfer of adult mosquitoes with a mouth aspirator and enable regular replacement of sugar water. Adult mosquitoes were provided permanent access to a 10% sugar solution by way of soaked cotton wool in a small plastic container. Male and female mosquitoes were kept together in these buckets for reproduction, and sample specimens were removed periodically for experimental purposes as required. For egg production, female mosquitoes were provided with a blood meal three time a week. Blood meals were provided by human volunteers placing their exposed arms against the netting at the top of the lid, mosquitoes then feeding through the netting. All mosquitoes in the colony were maintained in conditions that do not enable contamination with malaria or other parasites that could infect humans. There was therefore no risk of disease transmission to or between humans. For collection of eggs, small plastic trays with water were placed on the floor of the mosquito bucket-cages containing adults. Adult *An. arabiensis* were harvested from this colony for use in the cattle-feeding experiments and were generally obtained 2 to 5 days post-emergence from pupae. Adults that had been fed on treated and control cattle were treated as described below.

### Cattle treatment

**Fig. 1:**
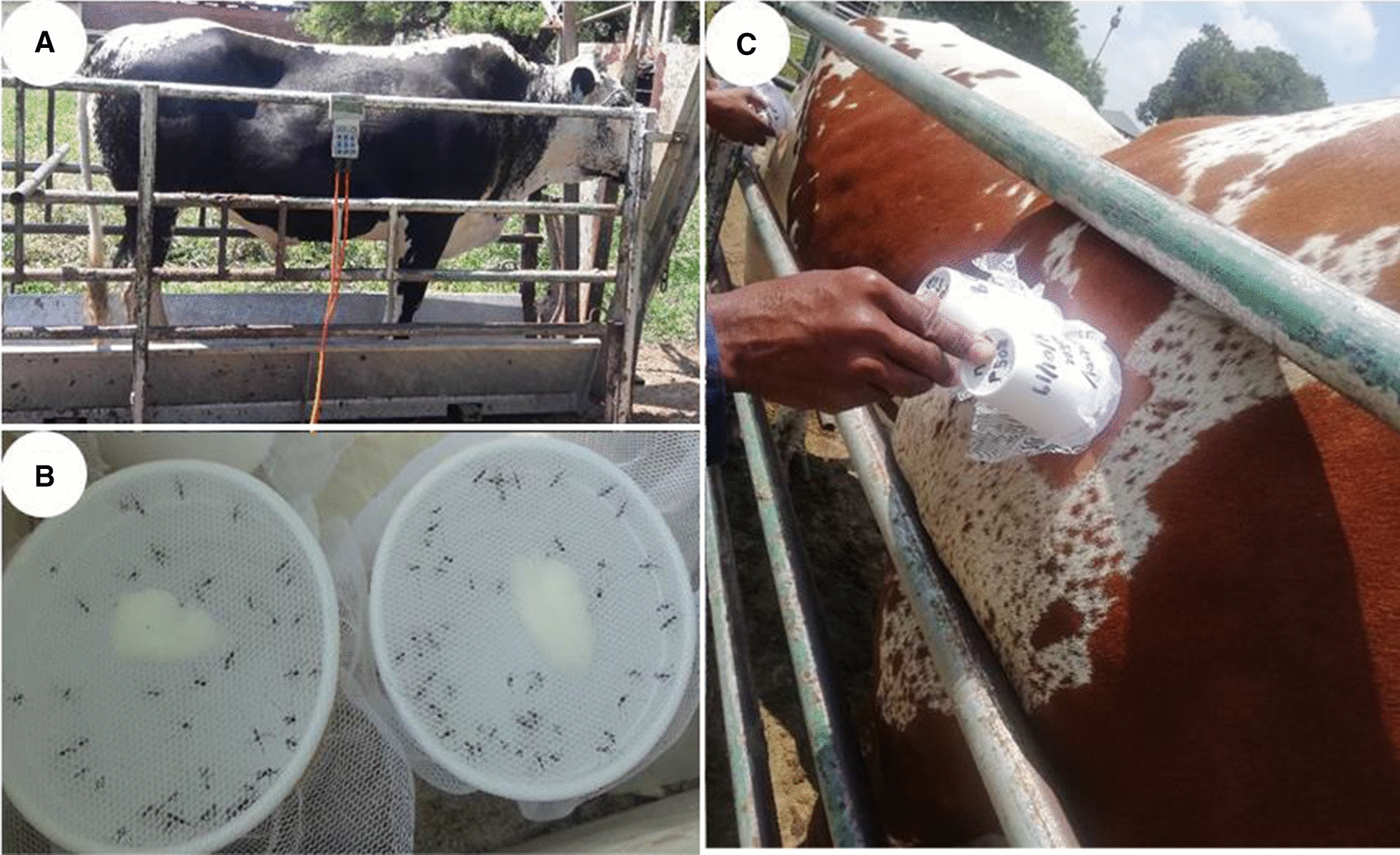
An overview of the mosquito blood-feeding experiments. **A** Cattle were kept in crushes and their weights were measured before treatment, **B** duplicate cups with 30 mosquitoes each covered by netting, **C** mosquitoes were blood-fed by applying the cups against the cattle on shaved spots for 15 min

Six cattle (Pinzyls strain, Nguni crossbreed with Pinzgauer) were housed and cared for at the Experimental farm of the University of Pretoria, Pretoria. This cattle strain is a cross-breed of the dominant cattle breed, Nguni, in South Africa’s malaria endemic areas [64]. In this facility, animals are kept outdoors in groups, where they graze freely and have permanent unimpeded access to water and shade. When required for experimental purposes, the animals are restrained in crushes (Fig. 1A) after which they are returned to their paddocks. None of these six cattle had been treated with any insecticides or acaricides for at least 3 months prior to the commencement of the experiments described herein. The weight of each experimental animal was determined (Fig. 1A) before initiation of treatment and found to range from 570 to 793 kg (see Appendix 1). Two endectocide drugs, namely 1% ivermectin (Noromectin®; Norbrook Laboratories, Centurion, South Africa) and 0.9% fipronil (Attila®; Ascendis Health, Sandton, South Africa), were used. We were unable to obtain injectable fipronil and hence opted for the pour-on formulation. Two of the cattle were treated with ivermectin (0.2 mg/kg body weight, subcutaneous injection), two with fipronil (1.0 mg/kg body weight, pour-on formulation) and the remaining two animals served as control (saline, applied through subcutaneous injection and as pour-on), with all six cattle receiving each of the treatments during the study period. Ivermectin and fipronil were administered at the respective manufacturer’s recommended dosages and applications. Fipronil was sprayed in two lines on both sides of the spinal cord from the base of the head to the tail root. Experiments were conducted in three replicates spaced 1 month apart to allow the applied drugs to be eliminated from the cattle or to decline to undetectable levels before the next trial. The order in which individuals received the treatments was randomised.

### Mosquito bioassays

Mosquito blood-feeding bioassays were conducted at days 0, 1, 4, 7, 13, 21 and 25 post-treatment. These days were chosen based on the pharmacokinetic profiles of injectable ivermectin and pour-on fipronil in cattle [61, 63]. Day 0 post-treatment represents the day each experimental cattle individual was treated; the feeding experiments were initiated 2–3 h after treatment. The days were standardised for pour-on fipronil to be the same as for ivermectin for comparative purposes. All mosquito exposure experiments were performed in duplicate for all experimental cattle individuals. Two circular areas, slightly larger than the size of a paper cup opening (70 mm), were shaved on all experimental cattle on the upper back. The removal of fur to facilitate mosquito feeding is commonly used in studies but may impact the topical insecticide's potency although not that of the injected insecticide. A mouth aspirator was used to transfer female mosquitoes (*n* = 30 mosquitoes per cup) to polystyrene cups (25 ml) covered by netting (Fig. 1B). Female mosquitoes were distinguished from the males by visual observation. The cups with mosquitoes were exposed to the shaved spots of each cattle individual for 15 min to allow adequate time for feeding (Fig. 1C). Different cups with new batches of mosquitoes were used for each feeding experiment.

After the feeding experiments, on each day post-treatment unfed mosquitoes were separated from the blood-fed specimens using a mouth aspirator after visual inspection. The abdomen of a blood-fed mosquito becomes distended and red in colour for several hours after feeding. Unfed mosquitoes were excluded from the experiments. Blood-fed mosquitoes were kept in polystyrene cups covered by netting for observation in the insectary and provided with permanent access to a 10% sugar source. Dead mosquitoes were counted and mortality was recorded once a day for successive days until the death of all mosquitoes from each cup.

### Egg production

Additional *An. arabiensis* mosquitoes were placed in six polystyrene cups (*n* = 10 mosquitoes per cup), and each cup was assigned and coded to a particular cattle individual for blood-feeding. The mosquitoes used for fecundity analysis fed from the cattle after the mosquitoes used for the survival analysis had fed from the same cattle. The blood-fed mosquitoes were placed individually into separate glass vials for egg production. Wet filter paper was placed at the bottom of each vial to encourage mosquitoes to lay eggs. The glass vials were covered with netting, and each mosquito was provided with a 10% sugar source. Mosquitoes were monitored for egg production on a daily basis. Filter papers in glass vials with mosquitoes that had laid eggs were placed under a dissecting microscope (Olympus SZ51; Olympus Corp., Tokyo, Japan) and the eggs counted daily from the first day the mosquito laid the eggs until her death. Eggs were destroyed after being counted to avoid double-counting. The proportion of *An. arabiensis* mosquitoes that laid eggs was used to determine the mean number of eggs for the three treatments.

### Statistical analysis

A non-parametric Kruskal–Wallis test was used to compare the proportion of mosquitoes that fed between treatments (control, ivermectin- or fipronil-treated). The survival data were not normally distributed (Kolmogorov–Smirnov test: 0.102,* df* = 252, *P* < 0.001). The effects of treatment (control, ivermectin or fipronil) on the survival of *An. arabiensis* were determined using the Cox proportional hazard model (coxph) package in the statistical software R version 3.6.1 (https://cran.r-project.org/bin/windows/base/old/3.6.1/). The coxph model is a semiparametric model that uses the hazard ratio to measure the risks between the treatments and control by comparing the survival curves. Included as independent variables were treatments, days post-treatment (i.e. days 0, 1, 4, 7, 13, 21 and 25) and days post-feeding (i.e. from day 1 until all mosquitoes died) as well as all two- and three-way interactions. The mortality of mosquitoes was the dependant variable. The cattle identity and replicate number (i.e. first, second or third) were included as random effects. The Kruskal–Wallis test was further used to investigate whether treatment had a significant effect on the proportion of mosquitoes that laid eggs. The data on *An. arabiensis* egg production were not normally distributed (Kolmogorov–Smirnov test: 0.229,* df* = 197, *P* < 0.001); therefore, this analysis was conducted using the generalised linear mixed model (GLMM) with a Poisson distribution that uses the log-link function to test the effect of treatment on the egg production of *An. arabiensis*. The number of eggs produced was the dependant variable while the treatments, days post-treatment and days post-feeding were the independent variables. The effect of treatment on the proportion of mosquitoes that laid eggs was also evaluated. This analysis was carried out using IBM SPSS version 25 (IBM Corp., Armonk, NY, USA) Results for both the survival and egg production sections are reported as means ± SE.

### Ethical clearance

Ethical clearance for use of cattle was obtained from the University of Pretoria Animal Ethics Review Committee (Ethics reference number: EC063-18, 180000035).

## Results

### Survival

Analysis to investigate the effects of ivermectin and fipronil on *An. arabiensis* survival was conducted on a total of 4940 mosquitoes blood-fed from the experimental cattle. Treatment did not significantly affect the proportion of mosquitoes that fed on cattle for the three treatments (Kruskal–Wallis H-test:* H* = 5.04,* df* = 2, *P* = 0.08). The overall proportion of mosquitoes that fed was 65, 64 and 67% for the ivermectin, fipronil and control cattle, respectively. The Cox proportional hazard model showed that treatment (*X*^2^ = 1182, *df * = 2, *P* < 0.001) had a significant effect on the survival of *An. arabiensis*. The mortality of *An. arabiensis* was 3.52-fold higher for ivermectin (*X*^2^ = 1082,* df * = 1, *P* < 0.001) and 2.43-fold higher for fipronil (*X*^2^ = 578.4, *df * = 1, *p* < 0.001) compared to the control group, respectively. Ivermectin increased mosquito mortality by a risk factor of 1.51 compared to fipronil (*X*^2^ = 133.7, *df * = 1, *P* < 0.001). The day post-treatment (*X*^2^ = 196.1, *df * = 6, *P* < 0.001) had significant effect on the mortality of *An. arabiensis*. Post-hoc comparisons showed significant effects at day 7, 13 and 21 post-treatment where the effects were 0.729, 0.769 and 0.762 higher than at day 0 post-treatment, respectively (*X*^2^ = 2746, *df * = 20, *P* < 0.001). Furthermore, the interaction between treatment and day post-treatment also significantly affected survival (*X*^2^ = 2746, *df * = 12, *P* < 0.001).

**Fig. 2:**
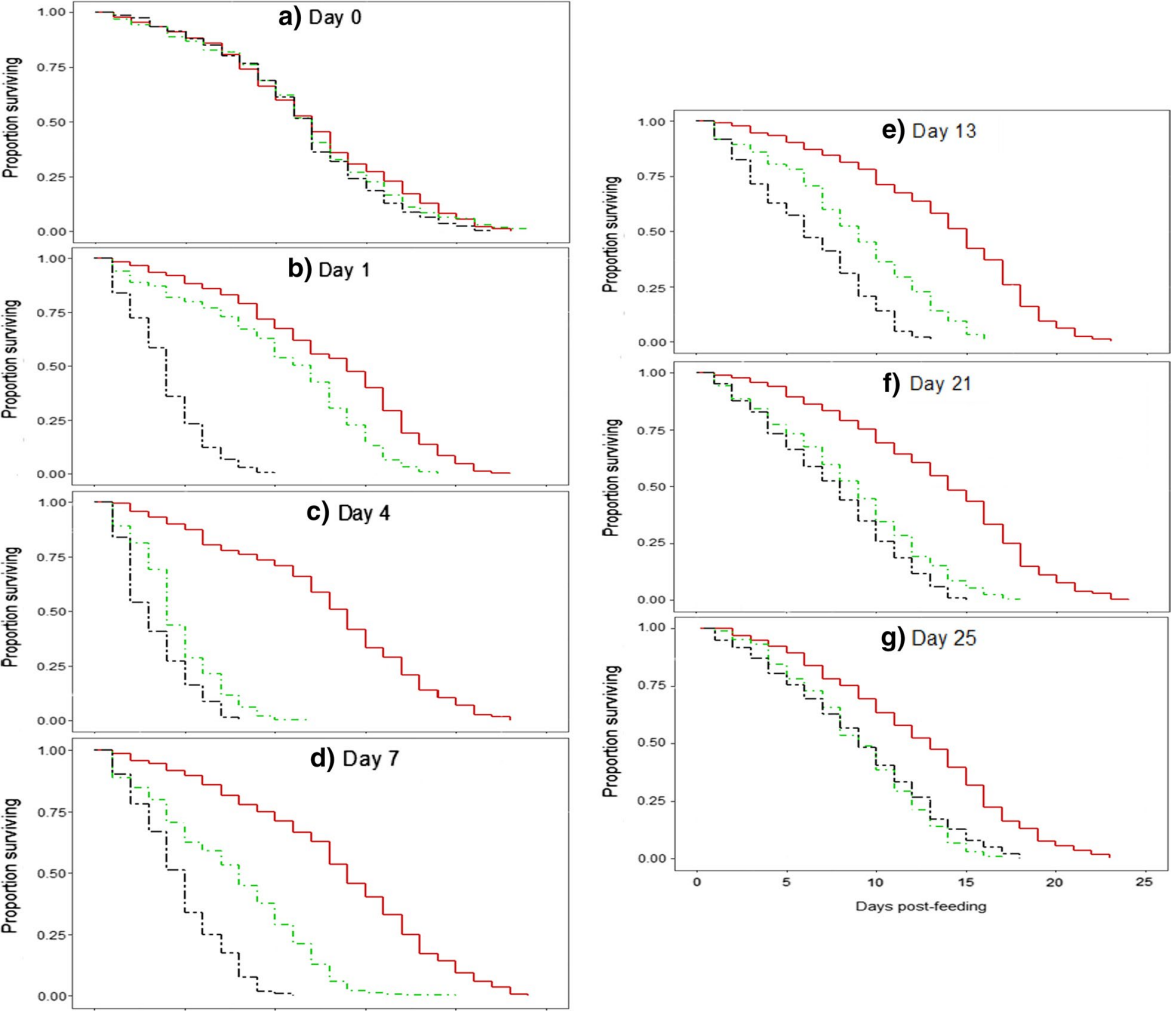
Estimates of *Anopheles arabiensis* survival after blood-feeding on control (red line) and ivermectin- (black line) and fipronil- (green line) treated cattle at different days post-treatment: **a** day 0, **b** day 1, **c** day 4, **d** day 7, **e** day 13, **f** day 21, **g** day 25. The lines represent survival curves from the Cox proportional hazard model regression

Treatment had no significant effect on the survival of *An. arabiensis* on day 0 post-treatment (*X*^2^ = 4.84, *df * = 2, *P* = 0.09; Fig. 2a). The effect of treatment was significant from day 1 post-treatment (*X*^2^ = 439.8, *df * = 2, *P* < 0.001, Fig. 2b). Ivermectin reduced the survival of *An. arabiensis* to a greater extent than fipronil from day 1 until day 21 post-treatment (Fig. 2b–f; Table 1). The mortality risk of *An. arabiensis* mosquitoes was highest for both ivermectin and fipronil at day 4 post-treatment with a hazard ratio of 18.49 ± 0.13 and 10.87 ± 0.15, respectively (Fig. 2c; Table 1). At day 4 post-treatment, the mortality of mosquitoes that fed on ivermectin-treated cattle was up to 60% higher than that of those that fed on the control group 4 days after exposure, with the former achieving a 100% mortality rate within 8 days. The mortality of mosquitoes that fed on fipronil-treated cattle reached 60% within 6 days and 100% within 10 days. From day 7 post-treatment onwards, the mortality of *An. arabiensis* that fed from the treated cattle gradually decreased and the hazards ratio was 4.67 ± 0.11 and 3.39 ± 0.11 at day 21 post-treatment for ivermectin and fipronil, respectively (Table 1). Although the treatment effect had gradually decreased at day 25 post-treatment, it was still significant (*X*^2^ = 75.68, *df * = 2, *P* < 0.001) with a difference of ≤ 20% between treatment and control.

**Table 1:** Comparison of the risks between the treatments (ivermectin and fipronil) and control at different days post-treatment

Days post-treatment	Treatment	Hazard ratio: exp(coeff) ± SE	95% Confidence interval (lower, upper)
0	Ivermectin	1.22 ± 0.09	1.02, 1.46
	Fipronil	1.06 ± 0.09	0.88, 1.27
1	Ivermectin	14.80 ± 0.13	11.47, 19.09
	Fipronil	2.02 ± 0.10	1.67, 2.45
4	Ivermectin	18.49 ± 0.16	13.64, 25.07
	Fipronil	10.87 ± 0.15	8.11, 14.56
7	Ivermectin	11.52 ± 0.13	8.94, 14.85
	Fipronil	4.17 ± 0.11	3.37, 5.17
13	Ivermectin	7.82 ± 0.12	6.16, 9.91
	Fipronil	3.82 ± 0.11	3.08, 4.76
21	Ivermectin	4.67 ± 0.11	3.76, 5.79
	Fipronil	3.39 ± 0.10	2.78, 4.15
25	Ivermectin	2.26 ± 0.11	1.83, 2.79
	Fipronil	2.59 ± 0.11	2.09, 3.21

*SE* Standard error

### Egg production

**Fig. 3:**
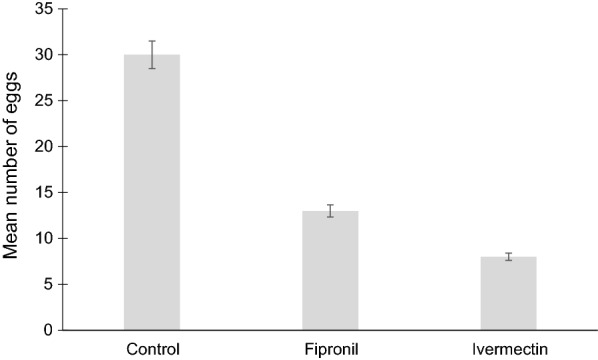
Effect of treatment on the number of eggs laid by *An. arabiensis*. Bars represent the mean ± standard error (SE)

Analysis for egg production was conducted on a total of 198 *An. arabiensis* mosquitoes that blood-fed from control and fipronil- and ivermectin-treated cattle. The proportion of mosquitoes that laid eggs was not significantly affected by treatment (Kruskal–Wallis H-test: *H* = 4.268,* df* = 2, *P* = 0.118) and was 46.67, 57.71 and 65.17% for the ivermectin, fipronil and control groups, respectively. The GLMM showed that treatment had a significant effect on the number of eggs laid (*F* = 49.98,* df* = 2, *P* < 0.001). Post-hoc comparisons showed that after feeding on cattle treated with ivermectin (7.53 ± 0.96 eggs;* t* = 6.835, *df* = 102, *P* < 0.001) and fipronil (12.46 ± 1.54 eggs; *t* = 5.798, *df* = 102, *P* < 0.001), the number of eggs laid by mosquitoes was significantly lower compared to the control group (29.88 ± 1.85 eggs; Fig. 3). There was no significant difference between the number of eggs laid by mosquitoes that fed on ivermectin and fipronil-treated cattle (*t* = 1.749, *df* = 102, *P* = 0.083). Overall, days post-treatment did not significantly affect the number of eggs produced (*F* = 1.75, * df* = 6, *P *= 0.118). However, the interaction between treatment and days post-treatment was significant (*F* = 2.59,* df* = 12, *P* = 0.005).

**Fig. 4:**
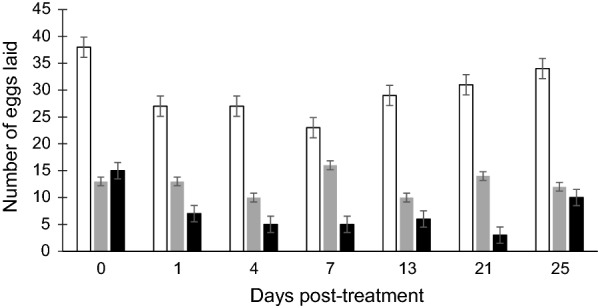
Estimates of mean number of eggs laid by *An. arabiensis* mosquitoes that blood-fed from control (open bars) and fipronil- (grey bars) and ivermectin-treated (black bars) cattle at 0, 1, 4, 7, 13, 21- and 25-days post-treatment. Bars represent the mean ± SE

The significant effect of treatment on the number of eggs laid was persistent throughout the days post-treatment from day 0 until day 25. At day 4 post-treatment, there was a significant effect for all comparisons amongst the three groups: ivermectin* versus* control (*t* = 4.10, * df* = 102, *P* < 0.001), ivermectin* versus* fipronil (*t* = 2.15,* df* = 102, *P* = 0.034) and fipronil* versus* control (*t* = 2.50,* df* = 102, *P* = 0.014). Both drugs significantly affected the number of eggs laid at day 7 (ivermectin: *t* = 3.08,* df* = 102, *P* = 0.003; fipronil:* F * = 2.94,* df* = 102, *P* = 0.004) and day 13 (ivermectin: *t* = 3.37, * df* = 102, *P* = 0.001; fipronil: *t* = 2.48, * df* = 102, *P* = 0.015) post-treatment (Fig. 4). All post-hoc comparisons for day 21 were significant: ivermectin* versus* control (*t* = 4.30,* df * = 102, *P* < 0.001), ivermectin *versus *fipronil (*t* = 3.30, *df* = 102, *P* = 0.001) and fipronil* versus* control (*t* = 2.04, * df* = 102,* P* = 0.044). The analysis for day 25 post-treatment showed a significant effect for ivermectin* versus* control (*t* = 3.45,* df* = 102, *P* = 0.001) and fipronil *versus* control (*t* = 2.91,* df* = 102, *P* = 0.005), but no significant effect between ivermectin and fipronil (*t* = 0.72,* df* = 102, *P* = 0.471) (Fig. 4).

Ivermectin led to a reduction in egg production by up to ± 90% while fipronil decreased egg production by up to ± 60% (Fig. 3). Comparison of days post-treatment showed significant differences in the overall number of eggs laid between day 4 and 25 (*t* = 2.405,* df* = 90, *P* = 0.018) and between day 7 and 25 (*t* = 2.709,* df* = 90, *P* = 0.008) (Fig. 4). At day 21 post-treatment, both ivermectin and fipronil were still highly effective (*F* = 9.70,* df* = 2, *P* < 0.008; Fig. 4). The post-hoc comparisons for interactions between treatment and days post-treatment showed significant effects at day 1 (*t* = 2.396, *P* = 0.019) and 21 (*t* = 2.331, *P* = 0.022) (Fig. 4). The effect of the treatment was still significant at day 25 post-treatment and reduced egg production by > 50% (*F* = 12.50,* df * = 2, *P* < 0.002; Fig. 4).

## Discussion

In the present study, we investigated the effect of two endectocides (ivermectin and fipronil) on the survival and fecundity of *An. arabiensis*. In accordance with the first prediction, the results demonstrated that both ivermectin and fipronil are able to reduce the survival of *An. arabiensis*. Mortality was increased by 77 and 70% with ivermectin and fipronil, respectively, compared to the control group, and additionally those mosquitoes that did survive the treatment exhibited a significantly reduced fecundity (to 90 and 60% for ivermectin and fipronil, respectively). The results for the effect of ivermectin on the survival of *An. arabiensis* found in this study are comparable to those reported for this species in other studies. Pooda et al. [20] reported a reduction in mortality of 75% in the third week post-treatment and 45% in the fourth week [20]. In a study by Lyimo et al. [36], the survival and fecundity of *An. arabiensis* were reduced by 52.5 and 64.6%, respectively [36]. In both of these studies [20, 36], invermectin was administered using the same subcutaneous injection administration method and at the same concentration as in the present study, and the treated cattle were also in a semi-field setting. Although Pooda et al. [20] used a sibling species of *An. arabiensis* (*Anopheles coluzzii*), the results for mortality are still comparable. Ivermectin has also been found to be effective in other livestock, such as pigs [65]. Pasay et al. [65] treated pigs with ivermectin to investigate its effect on *Anopheles farauti* survival and fecundity and reported reductions of 75 and 50%, respectively. In comparison to ivermectin, research into the use of fipronil for malaria control is limited. Dreyer et al. [46] investigated the impact of topical fipronil and injectable ivermectin on the survival of *An. albimanus* and reported results opposite to those of the present study. In their study, fipronil was found to be more effective than ivermectin at days 2, 5 and 7 post-treatment as it killed the *An. albimanus* at a faster speed than ivermectin. The observed opposite effects the Dreyer et al. study [46] and the present study suggest that the two drugs seem to affect the two mosquito species differently. Poché et al. [47] investigated the efficacy of oral fipronil against *An. arabiensis* in cattle and found that the mosquito indoor resting density (number of mosquitoes resting on walls and other surfaces inside houses) was reduced by 89%. Data on the overall effect of cattle- or livestock-administered fipronil on the fecundity of *Anopheles* mosquitoes could not be obtained from the literature. To the best of our knowledge, the present study is the first to measure the effect of fipronil on fecundity, with the finding that fipronil significantly reduced *An. arabiensis* fecundity; this result is comparable to the effects of other endectocides, ivermectin in particular, on *An. arabiensis* and other vectors.

As predicted for the second objective, injected ivermectin was more effective than the topical fipronil. The ivermectin treatment suppressed the survival of *An. arabiensis* quicker and this effect lasted for longer than the fipronil treatment although both chemicals were similarly effective during most of the measurement. As mentioned earlier, subcutaneous injections seem to yield better results than the topical or pour-on treatment. The different administration methods for ivermectin and fipronil are a limitation in this study because as a result we could not distinguish between the effects of the drug itself and the effect of the application methods. The application methods differ in their efficacy, and injected fipronil might produce better results. However, in a malaria endemic region, with higher numbers of livestock that would require treatment, the pour-on method could be a better option. The results of this study suggest that different routes of administration should be considered and that additional endectocides rather than just injectable ivermectin could also have the potential to be added into the malaria control toolbox. Oral [16, 47] and pour-on [46] fipronil have been found to be effective against malaria vectors (Table 1). In general, subcutaneous treatment has been shown to be more effective than oral and topical applications. Subcutaneous injection leads to a higher distribution of drugs and increases their duration of residence in lipids [20, 66]. Although injectable endectocides would be preferable due to their quick absorption, they are more costly to administer because they require needles and an experienced person [63]. The uptake of topical fipronil has been investigated previously by Cochet et al. [67]. Similar to what was observed in the present study, these authors reported a delay in uptake, with fipronil not being immediately absorbed from the site of application but translocated dermally and becoming confined in sebaceous glands and lipids of hair follicles. This may be a possible explanation of why fipronil showed its effectivity at a later stage than the injected ivermectin in our study.

The reduced-survival effect of both endectocides on mosquitoes lasted for a period of about 3 weeks, which was 1 week less than we had predicted for the third objective. The largest effect was at day 4 post-treatment for ivermectin and at day 7 post-treatment for fipronil. Ivermectin showed its efficacy from day 1 post-treatment while the fipronil only started being effective from day 4 post-treatment. Surprisingly, treatment effects on egg production were already apparent at day 0 post-treatment as opposed to effects on survival, possibly attributable to the time delay in feeding of mosquito batches used for survival and egg production. Although the effects of both endectocides had significantly decreased at day 25 post-treatment, fipronil had a larger effect on mosquito survival than ivermectin at this day post-treatment. The mosquitoes that were used for the egg production analysis were fed later after the ones used for survival analysis. The additional time elapsed between feeding of mosquitoes for survival and fecundity was different between the groups and could be one possible reason for the observed differences. The drugs might also have a greater and faster physiological effect on the fecundity of *An. arabiensis* than on its survival, which might not be surprising since the nutrients from the blood meals are incorporated into the eggs. There are many factors that affect the fecundity of mosquitoes, such as the source and size of the blood meal [68, 69]. Some control mosquitoes laid relatively small numbers of eggs compared to others, resulting in a lower than the expected mean number of eggs for the control group. It has been previously observed that in the field, some *Anopheles* mosquitoes require multiple blood meals to produce larger batches of eggs [70]. The effect of both ivermectin and fipronil on fecundity was persistent over the 25 days of the study period, which could be an advantage for malaria control. Unlike the present study, most studies that conducted a similar type of research so far did not investigate the effect of endectocides on fecundity at all the days post-treatment [16, 20]. The differences in the duration effect between the present study and these earlier studies might be due to various factors, such as the strains of the mosquito vector and the cattle breed. The different strains of *Anopheles* mosquitoes will develop resistance to endectocides in different ways [46, 71], and this variable should be considered before any implementation of the strategy.

The short duration effect of current-generation drugs has been noted previously, which brings into focus the question of the validity of cattle-administered endectocides for malaria control; thus, modifications might be necessary. Ivermectin was effective for a longer period in a study where slow-release ivermectin implant formulations were used [55]. In this study, *An. arabiensis* fed from cattle that had received subcutaneous high-dose ivermectin from slow-release implants; these implants significantly reduced mortality for up to 40 weeks compared to the control group [55, 72]. Ivermectin implants in livestock could serve as a long-lasting malaria outdoor strategy. However, it will be crucial to also determine their safety, cost and practicality of use in malaria endemic regions. The use of long-lasting endectocides in livestock, particularly cattle, could enhance agricultural production and lead to food security [73]. However, the overuse of endectocides may also lead to resistance in cattle and parasites, and hence be more detrimental than beneficial. Worms, ticks and other parasites are the main cause for disease and productivity loss in livestock [73]. The safety of cattle meat consumption should also be considered. Cattle treated with ivermectin and fipronil should not be slaughtered for human consumption within 28 and 105 days of treatment, respectively [74, 75]. Furthermore, ivermectin should not be administered in lactating cattle if the milk products are used for human consumption [74]. It is crucial for most endectocides to be administered at their safe doses and not frequently as this may lead to poor effects and sometimes to the death of the animals [76]. The use of IRS and LLINs in areas dominated by zoophagic vectors will not aid in malaria elimination. An integrated approach whereby various strategies are implemented for malaria control is required if malaria is to be eliminated [77, 78].

## Conclusion

This study shows that *An. arabiensis* mosquitoes exhibit increased mortality and reduced fecundity after feeding on cattle treated with ivermectin or fipronil at their respective manufacturer’s recommended dosages. Ivermectin used in this study proved to be a more effective endectocide than fipronil, possibly due to the difference in their application method. Both endectocides were only effective for a period of up to 3 weeks. This limited period of efficacy has resulted in some doubts in the literature about the practical value of their use. However, given the current lack of effective vector control tools against outdoor-biting mosquitoes, this method may still provide a realistic option for significantly impacting outdoor-biting vectors given the large number of mosquitoes known to feed on cattle at night. More advanced forms of endectocide administration to cattle, such as a slow-release formulation, could lead to higher and prolonged concentrations of endectocides in the blood. In addition, strategic use of these endectocides at the beginning of the malaria season when vector populations are low may have a significant impact on malaria incidence.

## Data Availability

The datasets that were used are available and can be made available by the corresponding author upon reasonable request.

## Appendix 1

### The weight of cattle individuals used in the experiment

**Table:**

Cattle ID.	Weight (kg)
P452	666
P445	626
P480	570
P508	793
P472	771
P443	658
